# Bridging divides or deepening gaps? ICT, quality of life, and the challenge of inclusive development at the intersection of healthcare and human development in South Asia

**DOI:** 10.1186/s12889-025-25210-w

**Published:** 2025-11-28

**Authors:** Minhas Akbar, Wenjin Tang, Ammar Hussain, Petra Poulova

**Affiliations:** 1 School of Economics and Management, Changsha University of Science and Technology, Changsha, 410004 China; 2 Department of Informatics and Quantitative Methods, Faculty of Informatics and Management, University of Hradec Kralove, Hradec Kralove, Czech Republic; 3 School of Management, University of Science and Technology of China, Hefei, China

**Keywords:** ICT, Healthcare expenditure, HDI, Quality of Life, Maternal Mortality, Out of pocket health expenditures, South Asia

## Abstract

**Background:**

The COVID-19 pandemic exposed critical gaps in healthcare infrastructure across developing countries, highlighting the need for scalable solutions to improve quality of life (QoL). This study investigates the asymmetric impact of Information and Communication Technologies (ICT) on Healthcare Expenditure (HCE) and the Human Development Index (HDI) across seven South Asian countries, where under-resourced healthcare systems coexist with rapid digital adoption.

**Methods:**

We use data from 2005 to 2023 and employ Fully Modified Ordinary Least Squares (FMOLS) and Dynamic Ordinary Least Squares (DOLS) techniques. ICT is measured through an aggregate index of mobile subscriptions, internet users and broadband connections.

**Results:**

The results reveal that: (1) ICT serves as a significant catalyst for aggregate Quality of Life, evidenced by positive effects on the HDI and HCE; (2) While ICT improves male human development (HDIM), its insignificant long run cointegration with female specific development (HDIF) confirms structural barriers persist despite aggregate progress in South Asia; (3) Moderation analysis highlights critical disparities, with ICT diffusion through male populations enhancing overall human development 7.5 percent more effectively than through female populations; (4) Urban contextualized ICT yields 7.3 percent stronger human development gains and 9.0 percent higher health expenditure impacts relative to rural diffusion; (5) Mediation analysis demonstrates that although ICT increases out of pocket health spending, these expenditures subsequently improve HDI; (6) ICT exhibits insignificant integration with maternal mortality, yet reduced maternal mortality independently elevates HDI.

**Conclusions:**

These findings necessitate gender- and geography-responsive ICT strategies, spatially balanced ICT infrastructure investment, and health financing safeguards to align South Asia’s technological transformation with sustainable development goals 3 and 5, ensuring digital dividends foster inclusive wellbeing.

## Introduction

The twenty-first century has been characterized by the remarkable growth of Information and Communication Technologies (ICT). It plays a crucial role in narrowing geographical barriers and making the world a global village. The pivotal role of ICT in every aspect of human life – including education, healthcare, commerce, product development, human development, energy consumption, economic development, environmental protection, and social interaction- can be seen in both developed and developing countries. However, this role is highly multifaceted and varies significantly across countries with varying levels of economic growth [1, 2]. In developed countries, ICT is a critical driver of automation, big data analytics, energy efficiency, smart cities, and high-tech industries [3]. In contrast, developing countries often leverage ICT primarily for foundational economic and social development, such as expanding digital literacy, improving access to essential services, and fostering inclusive financial systems through mobile banking and e-governance initiatives [4].

While this global dichotomy exists, South Asia, home to 2.03 billion people, presents a unique case among developing countries. Although ICT is experiencing a rapid growth in the region, critical gaps persist: the majority of the rural population lacks access to basic healthcare facilities, maternal mortality remains disproportionately high, and the average HDI (0.648) is lower than the global average (0.728) [5]. These disparities reflect unmet targets for Sustainable Development Goals 3 (health equity) and 5 (gender equality), particularly in underserved communities.

**Fig. 1:**
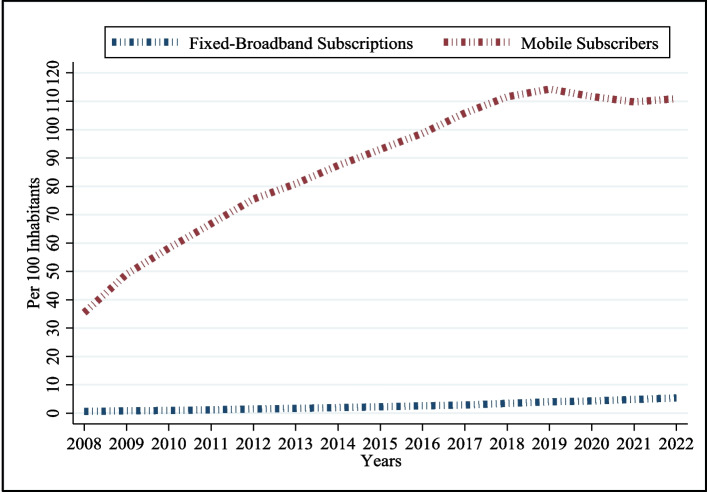
Variation in MPS and BBS per 100 individuals

**Fig. 2:**
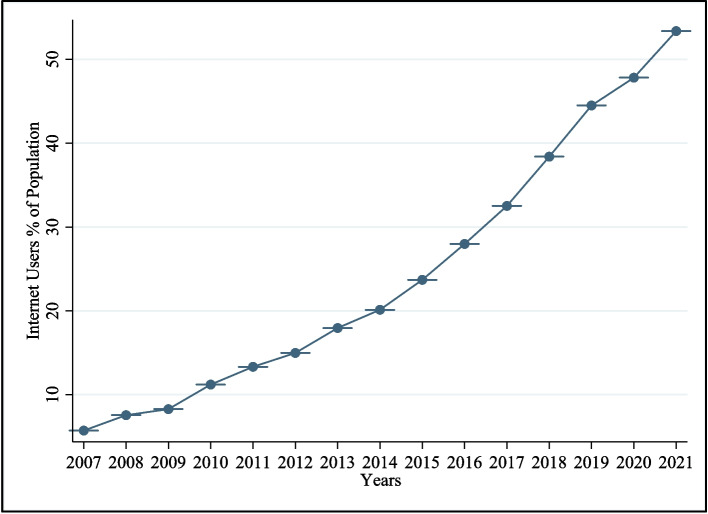
Variation in internet users’ % of population

Figures 1 and 2 reveal the past 15 years’ growth in some of the major ICT indictors. Figure 1 highlights the growth of fixed broadband and mobile phone subscribers. It shows that mobile subscriptions (per 100 individuals) were approximately 35 in 2008**,** but by the end of 2022, they reached 110. As illustrated in Fig. 2, the proportion of internet users relative to the total population has exhibited consistent growth, rising sharply from approximately 2% in 2007 to over 50% in 2021. However, the average internet penetration rate of the region remains relatively low at 53.8% [6], with pronounced urban–rural and male–female divides in digital access.

In developing economies, although ICT offer opportunities to improve social interaction, access to information, human development and healthcare, it also enables governments to deliver e-services. The existing literature has overly focused on examining the role of ICT infrastructure in financial development and economic growth [7–11]. However, the COVID-19 outbreak has put many economies under great stress, with developing countries being more prone to these negative effects due to weak healthcare infrastructure, limited financial reserves, and heavy dependence on sectors such as tourism and informal employment. These negative changes have turned the focus towards the overall quality of life of people in developing countries. According to Human Development Theory (HDT), income is only one element among various factors that are essential to fulfilling human needs. Nevertheless, the role of ICT extends beyond merely enhancing monetary income; it significantly contributes to the overall improvement in quality of life [1]. Therefore, the current study aims to explore the asymmetric impacts of ICT on the overall quality of life in South Asian nations.

Moreover, while extant literature predominantly examines ICT's aggregate healthcare impacts, it overlooks critical disparities in how benefits distribute across gender and geographic lines. The significance of this research lies in addressing this fundamental oversight in existing ICT-development literature: the failure to examine how technology's benefits distribute unevenly across gender and geography in developing economies. Current research attempts to examine how moderating factors like gender disparities (measured through male versus female population interactions) and geographic divides (urban versus rural population interactions) impact the role of ICT in shaping HCE and HDI. In addition, we also examine the mediating healthcare channels—particularly Out-of-Pocket Health Expenditures (OPHE) and Maternal Mortality (MM)—that transmit ICT's effects on wellbeing in regions with fragile health systems. When these variables are ignored, policymakers risk designing interventions that amplify inequality—for instance, digital health programs that benefit urban men (ICTUP/ICTMP) while excluding rural women (ICTRP/ICTFP), or ICT expansions that increase financial burdens (OPHE) without improving maternal survival (MM). By formally modelling these moderators and mediators, this study provides a realistic framework to steer digital transformation toward equitable outcomes in contexts where systemic gaps distort technological dividends.

More precisely, the study will attempt to answer the following questions: Is there a long-run relationship between ICT and QoL measures in South Asia? 2) does ICT improve QoL–in terms of HDI and healthcare expenditures-in the South Asian region? 3) Do these impacts differ for male (HDIM) versus female human development (HDIF)? 4) How do geographic ICT interactions (urban population vs. rural population) moderate HDI and healthcare outcomes? 5) How do gender-based ICT interactions (male population vs. female population) moderate HDI and healthcare outcomes? And 6) Do maternal mortality (MM) and out-of-pocket health expenditures (OPHE) mediate ICT’s impact on HDI?

Given that Quality of Life is a multidisciplinary concept spanning economics, sociology, political science, environmental science, and psychology [12]. It has been measured through various global indices, such as the OECD’s “Better Life Index”, the “Legatum Prosperity Index”, the “Human Development Index” and the “Well-being Index”. While each index comprises several factors- including income levels, education, personal well-being, health, and perception of the safety-the HDI remains the most widely trusted statistical measure for assessing QoL globally [13]. The HDI published by the United Nations Development Program (UNDP) incorporates four indicators: Gross National Income (GNI) per capita, life expectancy at birth, average years of schooling, and expected years of schooling. These indicators address three core dimensions of human life–health, education and living standards. Notably, HDI is stratified by gender (HDI male/HDI female), revealing systemic inequities in development outcomes.

**Fig. 3:**
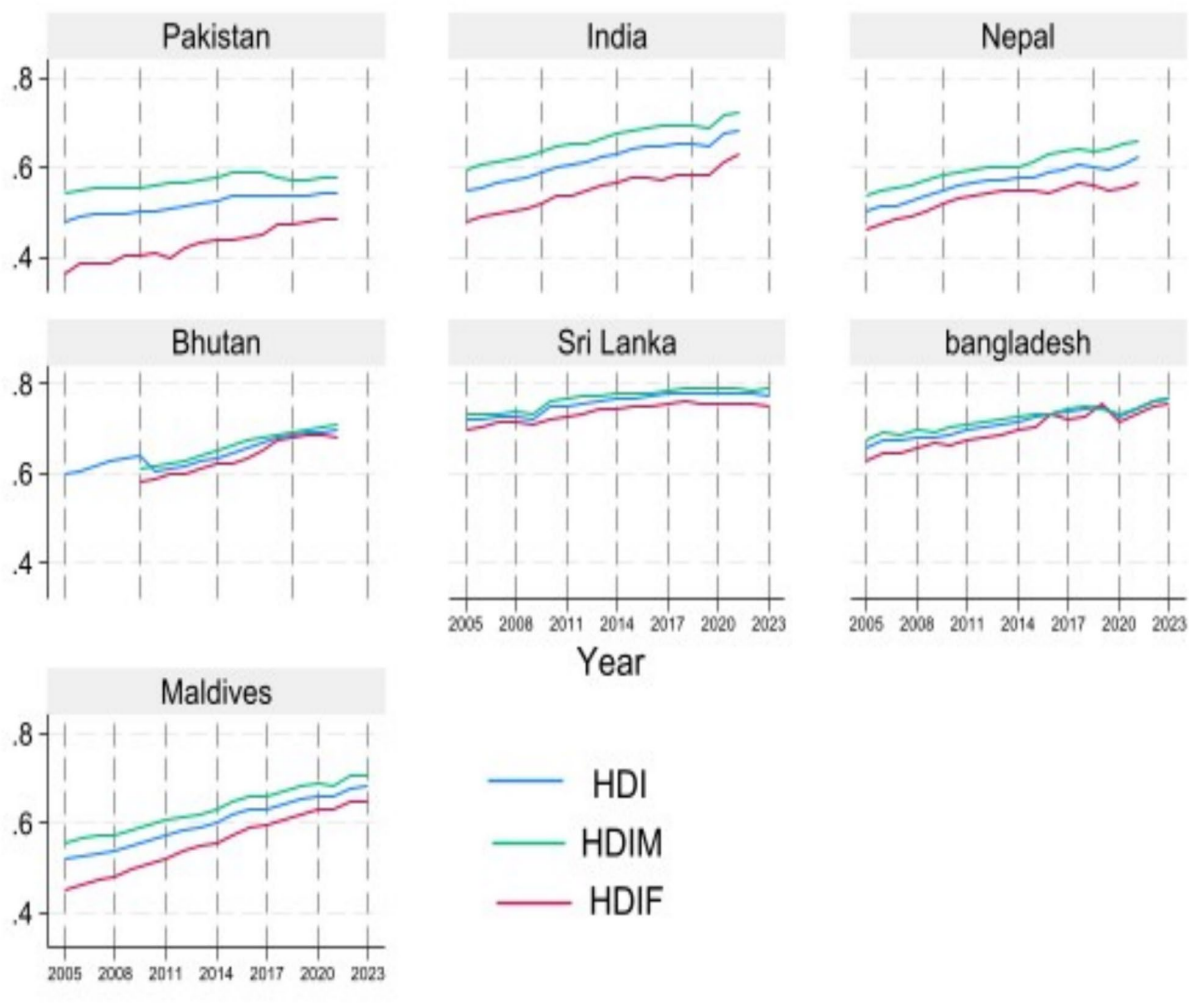
HDI variations over time

Figure 3 shows changes in HDI, male HDI (HDIM), and female HDI (HDIF) across selected South Asian countries from 2005 to 2023. The results reveal a consistent gap between HDIM and HDIF, with HDIF lower in every country. This disparity is especially pronounced in Pakistan, India, Bangladesh, and Nepal, while Bhutan, Sri Lanka, and the Maldives show relatively smaller gaps.

Healthcare-related infrastructure and facilities are a crucial factor in determining the quality of life of a population. Access to better healthcare facilities not only leads to increased life expectancy, higher productivity, improved health outcomes but also enhances overall QoL. Moreover, a positive bidirectional causality between healthcare expenditures and HDI has been reported by [14]. Additionally, several studies have proposed health-specific QoL measures [15–17], reinforcing that healthcare facilities, alongside HDI, play a vital role in improving a nation’s QoL. Thus, to assess ICT’s impact on the quality of life in South Asia, this study employs both the HDI and Healthcare Expenditures (HCE) as measures of the QoL.

**Fig. 4:**
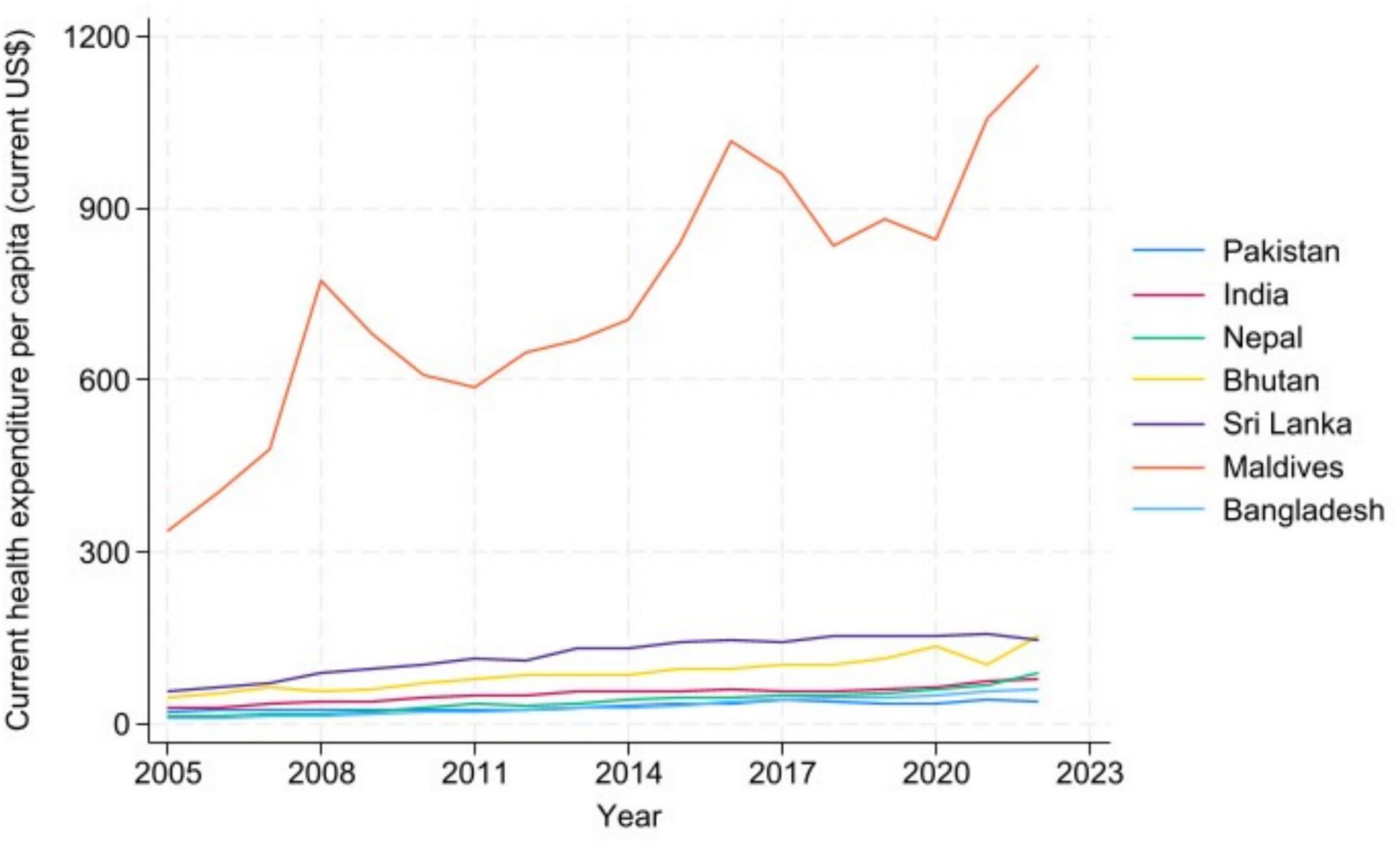
Healthcare expenditures over time

Figure 4 displays per capita healthcare expenditures across South Asian nations. The Maldives leads regional spending by a significant margin, investing substantially more in healthcare than any neighboring country. Sri Lanka ranks second, followed by Bhutan. Notably, apart from the Maldives, no South Asian nation has maintained an average annual healthcare expenditure exceeding $160 per capita during the study period.

**Fig. 5:**
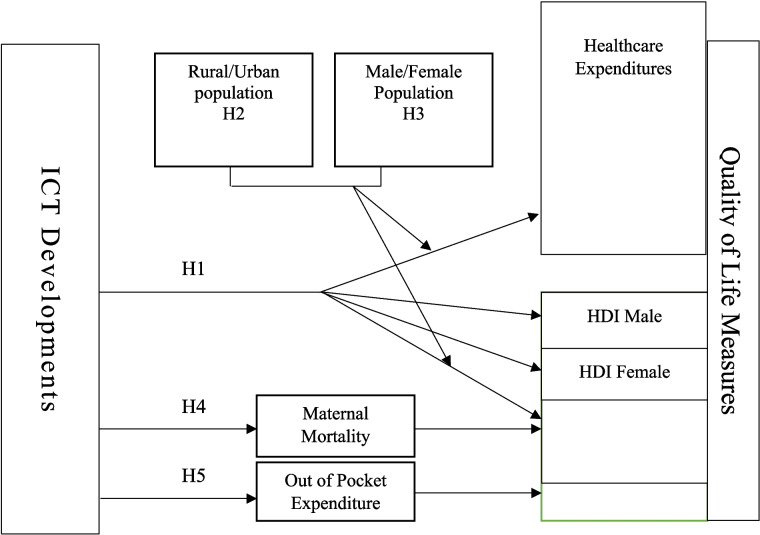
Theoretical framework

Figure 5 presents our study's theoretical framework. The analysis begins by examining the direct relationship between ICT (independent variable) and quality of life measures—healthcare expenditure (HCE) and human development index (HDI) (H1). Next, we assess how this relationship varies by population characteristics, testing urban versus rural residence (H2) and male versus female populations (H3) as moderators in the South Asian context. The framework then investigates the mechanisms linking ICT to HDI through two mediators: maternal mortality rates (H4) and out-of-pocket health expenditures (H5). Notably, we exclude HCE from the mediation analysis to prevent potential reverse causality issues, as both maternal mortality and out-of-pocket expenditures may themselves be influenced by healthcare spending levels.

While a handful of studies have explored the ICT-human development linkage [18–20], this study contributes to the literature in four ways. First, it focuses on South Asia, a region under-presented in existing studies. Second, unlike prior work that used limited ICT proxies (e.g., telecommunication investments) [18] or singular human development matrix [1, 19, 20], this study employs traditional digital infrastructure indicators (Mobile phone users, internet users, broadband connections and fixed telephone subscribers) to measure ICT Developments. Third**,** our moderation framework quantifies inequities in ICT returns, demonstrating urban populations (ICTUP) experience 7.3% stronger HDI gains than rural (ICTRP), and male-oriented diffusion (ICTMP) yields 7.5% higher HDI improvements than female (ICTFP)—providing empirical benchmarks for SDG 5 (gender equality) and SDG 9 (inclusive infrastructure) prioritization. Fourth, we pioneer mediation analysis revealing how out-of-pocket health expenditures (OPHE) channel ICT’s influence on HDI, exposing critical SDG 3.1–3.8 trade-offs. Further, we uniquely establish that unlike developed economies, ICT plays an insignificant role in reducing maternal mortality (MM) across South Asia. This critical finding challenges assumptions about digital healthcare efficacy and demands recalibrated approaches to achieve SDG 3.1’s maternal survival targets in resource-constrained settings. These divergent effects also underscore the need for region-specific digital strategies in developing economies.

The paper is structured as follows: The next section discusses the relevant literature and develops hypotheses; section three outlines the research design; Empirical results are presented in section four. The final section concludes the study with policy implications and future research agenda.

## Literature and signature theory

### Theory

The role of ICT in society and human behaviour has been theorized through multiple lenses, including the Technology Acceptance Model (TAM), Capability Approach Framework (CAF), Diffusion of Innovation theory, and Maslow’s Hierarchy of Needs [21–23]. Of these, the Capability Approach Framework (CAF) emerges as the most suitable for examining the relationship between technology and human development [24]. CAF provides a robust framework for assessing the impact of development initiatives-including ICT interventions-on targeted outcomes. Rooted in the principle of expanding individuals’ freedom, CAF conceptualizes human development as the enhancement of capabilities to achieve desired goals.

### ICT and quality of life

In contemporary society, ICT serves as a primary determinant in shaping human experiences. Numerous studies have examined the ramifications and implications of ICT on diverse facets of human life [25–30]. Bhuvana, Ramkumar [31] asserted that Covid-19 has underscored ICT’s critical role in healthcare domain. They emphasize on the significance of access to information as a key dimension for improving rural healthcare outcomes. Similarly, Anastasiadou, Tsipouras [32] argued that during crisis, digital health services fulfill a pivotal role as they enhance access to care, improve communication among providers, and deliver timely interventions.

Bhatti, Rafi [33] demonstrated ICT-enabled interventions for underserved population, particularly in empowering individuals with disabilities. A comprehensive examination of existing studies on ICT instruments and frameworks concluded that designed tools to meet the unique requirements of disabled migrants can cater to multiple dimensions, such as accessibility, communication, education, employment, and healthcare, thereby addressing the multifaceted challenges faced by this vulnerable group. Furthermore, Badran [34] highlights that ICT is harnessed in health-related products, services, and processes, which also includes the transformation of healthcare systems, the development of new skills, and the acquisition of new competencies in the healthcare system. This assimilation of ICT augments the overall economic and social value of healthcare, strengthening the well-being of citizens and enhancing the productivity and effectiveness of healthcare services. As ICT becomes increasingly available across different industries, it leads to efficiencies, and the healthcare sector is no exception. Electronic healthcare is an eminent implementation of ICT within e-health. It further indicates that ICT plays an imperative role in determining healthcare expenditures. Meena, Choi [35] found that sluggish lifestyles and evolving work environments have created challenges for global health and caused massive burdens on healthcare and fitness systems. These challenges can be addressed with the best physical practices and proper utilization of innovative technology.

Conversely, Shahzad, Jianqiu [36] found negative association between ICT and healthcare expenditures. A similar concurrent study reported negative connection between ICT and healthcare expenditures in the context of Saudi Arabia. The outcomes indicated that as ICT increases, healthcare expenditures tend to decrease, suggesting a potential cost-saving benefit associated with the implementation of ICT in the Saudi Arabian healthcare system [37]. Furthermore, although healthcare expenditures remain a critical determinant in enhancing a nation’s overall health [38], the association between spending levels and health outcomes is not linear. Increased health budgets do not invariably yield substantial improvements, particularly when resources are allocated inefficiently or inequitably [39].

Quality of life is an essential element that radically leverages the long-term growth of countries, regions, and cities. Despite its relevance, there are several issues in quantifying this concept, making it challenging to construct definitive guidelines on which economic components should be reviewed to improve living conditions. HDI operates as a comprehensive metric to assess a country's human development level among several dimensions, including health, life expectancy, and education. This index provides valuable insights into a nation's overall well-being and can inform policy-making decisions tailored to improving the quality of life for its citizens [40]. Thus, Zhang [41] investigated the association between ICT and HDI by examining data from Asian developing countries for 1990 to 2016; the study revealed that superior HDI scores were coupled with improved ICT infrastructure. These findings demonstrate the significance of investing in both ICT and human development initiatives to bolster overall growth and expansion in a nation. Another study by Gyamfi, Agozie [42] analysed the data of five South Asian countries from 1990 to 2016 and revealed that a trajectory improvement in ICT infrastructure could help these economies to raise the human development standards. In a similar vein, Ejemeyovwi, Osabuohien [43] found that ICT investment did not have any statistically significant impact on human development. The lack of ample influence from telecommunications investment on human development may be attributable to a combination of factors, such as relatively low investment in telecommunications and the high cost of technology acquisition. These factors may impede the potential effect that ICT could have on advancing human development, as limited resources and financial obstacles hinder the widespread adoption and implementation of technological advancements. It highlights the need to address these issues and examine the broader investments in ICT infrastructure and availability to stimulate positive response [44].

Ullah, Adebayo [45] argued that ICT is fully integrated with human life and very effective tool to address the modern world challenges. Moreover, ICT, captured by internet connections and mobile users significantly enhances human development. Machfud and Kartiwi [46], and Kowal and Paliwoda-Pękosz [47] also endorsed the existence of a strong and vivid association between ICT and HDI. Further, a recent study by Bala [48] found that new and contemporary measures of ICT significantly influence the human development standards but it also depends upon the country’s development stage and respective telecommunication services. Based on these evidences we propose following hypothesis;

H1: ICT positively influences the Quality-of-Life measures; HDI and HCE in South Asia.

### Moderating role of gender and geography

The distributional impacts of ICT across geographic and gender dimensions remain critically underexplored in developing economies. Urban–rural disparities in ICT infrastructure fundamentally moderate its developmental returns. Urban centres benefit from network effects [49] and healthcare digitization [50], enabling efficient resource allocation toward HDI and HCE. Conversely, rural areas encounter low digital skills, poor affordability and shortage of relevant content and applications [51, 52], constraining ICT's QoL benefits.

Similarly, gender-based moderation significantly shapes ICT outcomes. Patriarchal structures in South Asia restrict women's digital device ownership as GSMA reports 41% lower mobile ownership among women in South Asia [53]. while sociocultural norms limit skill development opportunities [54]. Consequently, ICT diffusion through male populations may disproportionately enhance economic participation and health service utilization. Further, these divides carry explicit SDG implications. Urban-centric ICT exacerbates spatial inequalities [55]. Based on these studies we put forward following hypotheses;

H2: ICT's impact on HDI and HCE is significantly stronger in urban populations than rural populations.

H3: ICT diffusion through male populations yields greater HDI and HCE improvements than through female populations.

### Mechanism analysis

ICT may influence Quality of Life through healthcare system mediators, yet these pathways remain largely ignored particularly in the context of developing economies. Therefore, we attempted to explore potential channels through which ICT may affect HDI. ICT enables teleconsultations for prenatal care [56], emergency obstetric coordination [57], and health worker training [58] that ultimately leads to reduced Maternal Mortalities (MM).

Simultaneously, ICT may reduce OPHE by streamlining administrative costs and enabling preventive care [59]. These savings could redirect household resources toward education and nutrition, indirectly boosting HDI. Thus, we propose following hypotheses;

H_4_: ICT reduces maternal mortality (MM), which subsequently enhances HDI.

H_5_: ICT lowers out-of-pocket health expenditures (OPHE), which in turn improves HDI.

## Research framework

### Data source

We use data from 7 South Asian countries (Bangladesh, Bhutan, India, Maldives, Nepal, Pakistan, and Sri Lanka) and exclude Afghanistan due to the unavailability of a considerable number of observations. We collect panel data on different variables for 2005 to 2023 from World Development Indicators (WDI), United Nations Development Project (UNDP) and Heritage Foundation.

### Model specification

To examine the impact of ICT on HCE and HDI, we employ panel data spanning the period from 2005 to 2023 for South Asian economies, we develop the following four models:

1 $$\begin{aligned}{HDI}_{i,t}= &{\gamma }_{0 }+{\gamma }_{1 }{ICT}_{i,t} +{\gamma }_{2 }{EF}_{i,t}\\&+{\gamma }_{3 }{INF}_{i,t}+{\gamma }_{4 }{GDP}_{i,t}\\&+{\gamma }_{5 }{POP}_{i,t}+{\mu }_{it}\end{aligned}$$

The first model examines the impact of ICT on Human Development Index (HDI) along with control variables. Descriptions of all variables are presented in Table 1.

**Table 1:** Variables Description

Symbol	Description	Source
HCE	Annual healthcare expenditures (per capita)	World Bank
HDI	Human Development Index	UNDP
HDIM	Human Development Index for male	UNDP
HDIF	Human Development Index for female	UNDP
ICT	PCA based composite index of ($IUI, MPS \& BBS$)	Authors Calculations
IUI	Internet users % of the population	World Bank
MPS	Per 100 subscribers of mobile phones	World Bank
BBS	Per 100 subscriptions of Fixed broadband	World Bank
MM	Maternal Mortality rate per 100,000 women	UNDP
OPHE	Out of Pocket expenditures by individuals	World Bank
ICTMP	ICT interacted with male population (% of total population)	Authors Calculations
ICTFP	ICT interacted with female population (% of total population)	Authors Calculations
ICTUP	ICT interacted with logged total urban population	Authors Calculations
ICTRP	ICT interacted with logged total rural population	Authors Calculations
EF	Economic freedom index	Heritage foundation
INF	Annual % of consumer prices inflation	World Bank
GDP	Natural log of net GDP	World Bank
POP	Natural log of annual population growth	World Bank

2 $$\begin{aligned}{HDIM}_{i,t}=& {\gamma }_{0 }+{\gamma }_{1 }{ICT}_{i,t} +{\gamma }_{2 }{EF}_{i,t}\\&+{\gamma }_{3 }{INF}_{i,t}+{\gamma }_{4 }{GDP}_{i,t}\\&+{\gamma }_{5 }{POP}_{i,t}+{\mu }_{it}\end{aligned}$$

Next, we construct model three to investigate the influence of the ICT index on the Human Development Index of male (HDIM).

3 $$\begin{aligned}{HDIF}_{i,t}= &{\gamma }_{0 }+{\gamma }_{1 }{ICT}_{i,t} +{\gamma }_{2 }{EF}_{i,t}\\&+{\gamma }_{3 }{INF}_{i,t}+{\gamma }_{4 }{GDP}_{i,t}\\&+{\gamma }_{5 }{POP}_{i,t}+{\mu }_{it}\end{aligned}$$

Model 3 assesses the effects of ICT index on the Human Development Index of female (HDIF).

4 $$\begin{aligned}{HCE}_{i,t}=& {\gamma }_{0 }+{\gamma }_{1 }{ICT}_{i,t} +{\gamma }_{2 }{EF}_{i,t}\\&+{\gamma }_{3 }{INF}_{i,t}+{\gamma }_{4 }{GDP}_{i,t}\\&+{\gamma }_{5 }{POP}_{i,t}+{\mu }_{it}\end{aligned}$$

The fourth model is designed to test the influence of the ICT index on healthcare expenditures, controlling for additional variables.

#### Heterogeneity effects

To examine the asymmetric effects of ICT on HCE and HDI across gender and geographical boundaries. We have developed eight econometric models

5 $$\begin{aligned}{HCE}_{i,t}=& {\gamma }_{0 }+{\gamma }_{1 }{ICT*MP}_{i,t} +{\gamma }_{2 }{EF}_{i,t}\\&+{\gamma }_{3 }{INF}_{i,t}+{\gamma }_{4 }{GDP}_{i,t}\\&+{\gamma }_{5 }{POP}_{i,t}+{\mu }_{it}\end{aligned}$$

6 $$\begin{aligned}{HCE}_{i,t}=& {\gamma }_{0 }+{\gamma }_{1 }{{ICT*FP}_{i,t}}_{i,t} \\&+{\gamma }_{2 }{EF}_{i,t}+{\gamma }_{3 }{INF}_{i,t}\\&+{\gamma }_{4 }{GDP}_{i,t}+{\gamma }_{5 }{POP}_{i,t}\\&+{\mu }_{it}\end{aligned}$$

7 $$\begin{aligned}{HDI}_{i,t}=& {\gamma }_{0 }+{ICT*MP}_{i,t} +{\gamma }_{2 }{EF}_{i,t}\\&+{\gamma }_{3 }{INF}_{i,t}+{\gamma }_{4 }{GDP}_{i,t}\\&+{\gamma }_{5 }{POP}_{i,t}+{\mu }_{it}\end{aligned}$$

8 $$\begin{aligned}{HDI}_{i,t}=& {\gamma }_{0 }+{\gamma }_{1 }{{ICT*FP}_{i,t}}_{i,t} \\&+{\gamma }_{2 }{EF}_{i,t}+{\gamma }_{3 }{INF}_{i,t}\\&+{\gamma }_{4 }{GDP}_{i,t}+{\gamma }_{5 }{POP}_{i,t}\\&+{\mu }_{it}\end{aligned}$$

9 $$\begin{aligned}{HCE}_{i,t}=& {\gamma }_{0 }+{\gamma }_{1 }{ICT*UP}_{i,t} \\&+{\gamma }_{2 }{EF}_{i,t}+{\gamma }_{3 }{INF}_{i,t}\\&+{\gamma }_{4 }{GDP}_{i,t}+{\gamma }_{5 }{POP}_{i,t}\\&+{\mu }_{it}\end{aligned}$$

10 $$\begin{aligned}{HCE}_{i,t}=& {\gamma }_{0 }+{\gamma }_{1 }{ICT*RP}_{i,t} +{\gamma }_{2 }{EF}_{i,t}\\&+{\gamma }_{3 }{INF}_{i,t}+{\gamma }_{4 }{GDP}_{i,t}\\&+{\gamma }_{5 }{POP}_{i,t}+{\mu }_{it}\end{aligned}$$

11 $$\begin{aligned}{HDI}_{i,t}=& {\gamma }_{0 }+{\gamma }_{1 }{ICT*UP}_{i,t} +{\gamma }_{2 }{EF}_{i,t}\\&+{\gamma }_{3 }{INF}_{i,t}+{\gamma }_{4 }{GDP}_{i,t}\\&+{\gamma }_{5 }{POP}_{i,t}+{\mu }_{it}\end{aligned}$$

12 $$\begin{aligned}{HDI}_{i,t}=& {\gamma }_{0 }+{\gamma }_{1 }{ICT*RP}_{i,t} +{\gamma }_{2 }{EF}_{i,t}\\&+{\gamma }_{3 }{INF}_{i,t}+{\gamma }_{4 }{GDP}_{i,t}\\&+{\gamma }_{5 }{POP}_{i,t}+{\mu }_{it}\end{aligned}$$

We develop Model 5–8 to examine the moderating role of female (FP) and male population (MP) between ICT developments quality of life measures, HCE and HDI. Similarly, we construct model 9–12 to examine moderating role of urban (UP) and rural population (RP) between ICT, HCE and HDI.

#### Mechanism analysis

To examine the mechanism through which ICT may influence HDI in South Asian countries. We have developed following four models.

13 $$\begin{aligned}{MM}_{i,t}=& {\gamma }_{0 }+{\gamma }_{1 }{ICT}_{i,t} +{\gamma }_{2 }{EF}_{i,t}\\&+{\gamma }_{3 }{INF}_{i,t}+{\gamma }_{4 }{GDP}_{i,t}\\&+{\gamma }_{5 }{POP}_{i,t}+{\mu }_{it}\end{aligned}$$

14 $$\begin{aligned}{HDI}_{i,t}=& {\gamma }_{0 }+{\gamma }_{1 }{MM}_{i,t}+{\gamma }_{2 }{ICT}_{i,t} \\&+{\gamma }_{2 }{EF}_{i,t}+{\gamma }_{3 }{INF}_{i,t}\\&+{\gamma }_{4 }{GDP}_{i,t}+{\gamma }_{5 }{POP}_{i,t}+{\mu }_{it}\end{aligned}$$

15 $$\begin{aligned}{OPHE}_{i,t}=& {\gamma }_{0 }+{\gamma }_{1 }{ICT}_{i,t} +{\gamma }_{2 }{EF}_{i,t}\\&+{\gamma }_{3 }{INF}_{i,t}+{\gamma }_{4 }{GDP}_{i,t}\\&+{\gamma }_{5 }{POP}_{i,t}+{\mu }_{it}\end{aligned}$$

16 $$\begin{aligned}{HDI}_{i,t}=& {\gamma }_{0 }+{\gamma }_{1 }{OPHE}_{i,t}+{\gamma }_{2 }{ICT}_{i,t} \\&+{\gamma }_{2 }{EF}_{i,t}+{\gamma }_{3 }{INF}_{i,t}\\&+{\gamma }_{4 }{GDP}_{i,t}+{\gamma }_{5 }{POP}_{i,t}+{\mu }_{it}\end{aligned}$$

Model 13 examines the impact of ICT on Maternal Mortality (MM) rate, while model 14 is designed to examine the subsequent impact of MM on HDI. Finally, model 15 captures the role of ICT in determining out of pocket expenditure (OPHE), and model 16 examines how these OPHE shape HDI in South Asia.

The widespread discourse on ICT in both academic and general literature underscores its broad-spectrum influence. In this study, we construct a composite ICT index using three key indicators: mobile phone subscribers, fixed broadband subscriptions, and internet users. The index is calculated using the following equation.

17 $$PCA=\overset3{\underset{i=1}{\sum\eta_{ij}}}\frac{X_{ij}}{SD_i}$$

where $PCA$ illustrates the composite index of three ICT indicators; ${X}_{ij}$ denotes the i^th^ variable at j^th^ year; $SD$ represents the standard deviation, and $\eta_{ij}$. Exhibits the factor load captured through PCA.

Results of our PCA have been reported in Appendix A1 and A2. The first component had an eigenvalue of 2.42, explaining 80.9% of the total variance in the three variables. No other components met the Kaiser criterion (eigenvalue > 1). The factor loadings, which represent the correlation between each variable and the principal component, are IUI (0.57), BBS (0.57), and MPS (0.58). It means that all three variables (IUI, BBS, MPS) contribute almost equally to the ICT development in South Asia. Moreover, the index was constructed using the scoring coefficients derived from the PCA. The resulting ICT development index is a weighted linear combination of the standardized variables, with weights proportional to their factor loadings on the first principal component.

### Methods

The primary objective of our study is to investigate the long-run relationship among our three variables: ICT, HC, and HDI. In the preliminary analysis, we first assess the order of integration of the data. To examine the stationary properties of the panel data series, we employed two widely used unit root test: the Im, Pesaran, and Shin [60] test and the Levin, Lin, and Chu [61] test. The following equation is used to run unit root tests;

18 $$-2 \sum_{i=1}^{n}\text{log}\left({\mu }_{1}\right) \to {X}_{2}^{2} n$$

These tests based on the null hypothesis "There is a unit root problem," and rejecting H_o_ corroborates the absence of a unit root issue. Once it is confirmed that all the proposed data series are at the same level of integration, the subsequent step is to investigate cointegration among the sampled variables. If there is a linear combination of the two non-stationary series, they are acknowledged to be cointegrated. The Kao cointegration [62] test is employed for the examination of cointegration.

The following example illustrates how the panel cointegration works:

$${\rm X}_{i,t}={\lambda }_{i,t}+{\gamma }_{i,t}\beta +{\mu }_{i,t}$$

where $i$ = 1, ….*Ν, t* = 1, β is the slope parameter, and ${\mu }_{i,t}$ stationary distribution, ${\chi }_{i,t}$ and ${\gamma }_{i,t}$ the integrated process of order I [1] for all *i.*

With cointegration established, the analysis proceeds to estimate the long-run coefficients. Given that all variables are integrated of order one, I [1], the Panel Fully Modified Ordinary Least Squares (FMOLS) technique is the most appropriate method. FMOLS is explicitly designed for such cointegrated systems, providing efficient estimates that are corrected for common statistical issues, and is therefore selected over other methods like ARDL or PMG.. Substantial empirical evidence advocates for the FMOLS estimator to robustly quantify long-run relationships [7, 63]. This method furnishes asymptotically long-run unbiased estimates and presents several additional advantages, including allowances for serial correlation (SE), endogeneity (EE), and cross-section heterogeneity. FMOLS achieves this through a non-parametric modification of the standard OLS procedure, incorporating the long-run covariance matrix to account for correlations between regressors and the error term, thereby yielding reliable standard errors for inference.

Moreover, to ensure the robustness and reliability of our long-run coefficient estimates, we complement the FMOLS estimator of [64] with the Dynamic Ordinary Least Square (DOLS) estimator proposed by [65] and [66]. While both estimators are designed to provide efficient and asymptotically unbiased estimates of cointegrating vectors in the presence of endogenous regressors and serial correlation, they employ fundamentally different strategies to achieve this goal. The FMOLS technique is a non-parametric approach. It applies a sophisticated correction to the standard OLS estimator after the initial estimation. In contrast, the DOLS estimator is a parametric approach. It specifically models the sources of bias within the regression equation itself. This is achieved particularly by augmenting the cointegrating regression with leads and lags of the first-differenced regressors.

## Empirical findings

### Summary statistics

Table 2 presents the country-wise summary statistics of variables, revealing substantial heterogeneity in developmental indicators across South Asian nations. Sri Lanka demonstrates the region's highest human development attainment with an average HDI of (0.76), closely followed by Maldives (0.71) and Bhutan (0.64), while Pakistan trails significantly at (0.52). Further, gender disparities emerge as particularly acute in Pakistan, where the HDIF-HDIM gap (0.43 vs. 0.57) exceeds regional counterparts, whereas Bhutan demonstrates near-parity (0.64 vs. 0.66). Health expenditure patterns exhibit striking divergence: Maldives reports extraordinary mean HCE of $748.9, followed by Sri Lanka's $120.56 and Bhutan $88.83, whereas Pakistan ($31.08) and Bangladesh ($31.80) manifest substantially lower investment. Financial burdens in healthcare are notably elevated in Bangladesh (OPHE = 69.69%) followed by Pakistan (62.27) and India (61.5), while Bhutan exhibit lowest OPHE (18.24%). Within the maternal mortality (MM) landscape, Nepal grapples with the region’s most severe burden, recording 263.18 maternal deaths per 100,000 live births. This figure substantially exceeds the rates observed in Bangladesh (236.86) and Pakistan (205.89), while India demonstrates moderate outcomes (158.17). Bhutan (96.01) and Maldives (58.70) reflect progressive improvements in maternal healthcare. Notwithstanding these variations, Sri Lanka achieves exceptional reproductive health outcomes (33.73) – a performance that contrasts markedly with the regional average of 150.36. This divergence underscores critical healthcare system disparities. Contextualized against Sustainable Development Goal (SDG) 3, which mandates reducing the global maternal mortality ratio (MMR) below 70 per 100,000 births with no nation exceeding twice this benchmark (140), the prevailing data reveal profound challenges. Current trajectories suggest most South Asian states substantially exceed the threshold, necessitating accelerated interventions beyond existing health system reforms to meet the 2030 target.

**Table 2:** Country-wise summary statistics

Pakistan					
**Variables**	**N**	**Mean**	**Sd**	**min**	**max**
HCE	18	31.08	7.42	19.59	42.8
HDI	19	.52	0.02	.48	.54
HDIM	19	.57	0.01	.55	.59
HDIF	19	.43	0.04	.37	.49
ICT	19	-.61	0.13	-.81	-.43
IUI	19	12.56	6.46	6.33	27.4
MPS	19	56.52	19.44	7.28	79.11
BBS	19	.71	0.46	.01	1.36
FTS	19	2.07	0.83	1.04	3.16
MM	19	205.89	42.46	154.16	300.75
OPHE	18	62.27	7.12	47.37	72.84
ICTFP	19	−29.79	6.21	−39.25	−21.06
ICTMP	19	−31.29	6.74	−41.66	−21.74
ICTUP	19	−11.07	2.26	−14.49	−7.86
ICTRP	19	−11.43	2.37	−15.02	−8.07
EF	19	54.46	2.38	48.8	57.9
INF	19	10.73	6.79	2.53	30.77
GDP	19	27.62	0.21	27.29	27.93
POP	19	1.94	0.46	1.3	2.68
India					
HCE	18	51.84	14.48	27.5	79.52
HDI	19	.62	0.04	.55	.69
HDIM	19	.66	0.04	.6	.72
HDIF	19	.55	0.04	.48	.63
ICT	17	-.52	0.16	-.78	-.15
IUI	17	15.98	14.70	2.39	55.9
MPS	19	62.67	26.28	7.81	85.97
BBS	19	1.18	0.69	.12	2.75
FTS	19	2.38	0.79	1.43	4.35
MM	19	158.17	56.40	102.65	285.73
OPHE	18	61.52	9.06	45.11	73.15
ICTFP	17	−24.91	7.84	−37.57	−7.08
ICTMP	17	−26.67	8.43	−40.28	−7.55
ICTUP	17	−10.22	3.17	−15.29	−2.94
ICTRP	17	−10.61	3.33	−15.98	−3.02
EF	19	54.51	1.21	52.2	56.5
INF	19	6.72	2.54	3.33	11.99
GDP	19	29.7	0.34	29.13	30.22
POP	19	1.24	0.25	.79	1.63
Nepal					
HCE	18	40.06	20.00	13.68	88.27
HDI	19	.57	0.04	.5	.62
HDIM	19	.6	0.04	.54	.66
HDIF	19	.53	0.03	.46	.57
ICT	17	-.36	0.48	-.81	.55
IUI	19	20.12	18.02	.83	55.8
MPS	18	76.11	52.12	.86	139.53
BBS	17	1.6	1.71	0	4.83
FTS	18	2.78	0.35	1.84	3.08
MM	19	263.18	73.59	174.41	380.39
OPHE	18	56.4	4.91	42.5	63.53
ICTFP	17	−18.24	24.71	−40.79	28.7
ICTMP	17	−17.59	23.55	−40.25	26.78
ICTUP	17	−5.47	7.44	−12.34	8.69
ICTRP	17	−6.07	8.18	−13.72	9.41
EF	19	52.18	1.81	49.7	55.1
INF	19	7.18	2.45	2.27	11.09
GDP	19	25.33	0.25	24.94	25.7
POP	19	.7	0.52	-.07	1.93
Bhutan					
HCE	18	88.83	28.32	46.79	154.49
HDI	19	.64	0.03	.6	.7
HDIM	14	.66	0.03	.61	.71
HDIF	14	.64	0.04	.58	.69
ICT	16	-.35	0.31	-.73	.25
IUI	19	38.94	31.75	3.85	88.4
MPS	19	70.82	31.31	5.44	100.33
BBS	16	1.62	1.10	.3	3.76
FTS	19	3.41	0.77	2.34	4.99
MM	19	96.01	42.78	57.32	185.8
OPHE	18	18.24	4.88	13.16	34.57
ICTFP	16	−16.35	14.36	−33.57	11.67
ICTMP	16	−18.96	16.65	−39.09	13.6
ICTUP	16	−4.44	3.90	−9.01	3.17
ICTRP	16	−4.6	4.04	−9.47	3.29
EF	15	58.62	2.22	55	62.9
INF	19	5.95	2.29	2.72	10.92
GDP	19	22.82	0.29	22.23	23.17
POP	19	1	0.27	.66	1.73
Sri Lanka					
HCE	18	120.56	33.47	55.4	157.13
HDI	19	.76	0.02	.72	.78
HDIM	19	.77	0.02	.73	.79
HDIF	19	.74	0.02	.7	.76
ICT	19	.3	1.01	-.78	2.17
IUI	19	23.1	15.27	1.79	51.2
MPS	19	97.54	40.16	16.63	151.73
BBS	19	3.93	3.56	.1	10.54
FTS	19	12.65	3.23	6.15	17.2
MM	19	33.73	4.96	28.84	44.01
OPHE	18	47.33	4.31	40.22	53.3
ICTFP	19	15.56	51.74	−39.67	111.87
ICTMP	19	14.34	48.84	−38.54	105
ICTUP	19	4.58	15.30	−11.83	33.06
ICTRP	19	5.01	16.78	−13	36.23
EF	19	57.52	2.40	52.2	61
INF	19	10.24	10.93	2.14	49.72
GDP	19	26.22	0.25	25.74	26.49
POP	19	.49	0.89	−2.8	1.14
Maldives					
HCE	18	748.9	223.39	335.14	1150.69
HDI	19	.71	0.03	.66	.77
HDIM	19	.72	0.03	.68	.77
HDIF	19	.7	0.04	.63	.76
ICT	19	1.32	1.45	-.51	4.48
IUI	19	49.06	26.43	6.87	84.7
MPS	19	142.78	33.08	66.45	196.41
BBS	19	7.55	5.14	1.06	18.73
FTS	19	6.18	3.27	2.44	14
MM	19	58.7	9.38	45.81	78.76
OPHE	18	29.9	12.67	14.92	55.66
ICTFP	19	51.5	55.04	−24.25	169.95
ICTMP	19	80.74	90.28	−26.81	278.42
ICTUP	19	16.12	17.85	−5.9	55.17
ICTRP	19	16.63	18.35	−6.24	56.62
EF	15	51.02	2.92	46.6	56.5
INF	19	3.71	4.00	−1.37	12.04
GDP	19	22.74	0.28	22.18	23.18
POP	19	2.93	0.87	.36	3.54
Bangladesh					
HCE	18	31.8	16.13	11.31	61.09
HDI	19	.6	0.05	.52	.69
HDIM	19	.63	0.05	.56	.71
HDIF	19	.56	0.07	.45	.65
ICT	17	.08	0.79	-.8	1.42
IUI	19	16.31	15.40	.24	44.5
MPS	19	69.63	35.40	6.22	114.36
BBS	17	3.14	2.81	.03	7.89
FTS	19	.63	0.25	.17	.9
MM	19	236.86	93.14	123.03	376.97
OPHE	18	69.69	2.62	65.38	74
ICTFP	17	4.08	40.17	−40.1	72.14
ICTMP	17	3.62	39.31	−40.26	69.86
ICTUP	17	1.49	14.19	−14.1	25.64
ICTRP	17	1.41	14.67	−14.84	26.19
EF	19	52.41	3.48	44.2	56.5
INF	19	7.1	1.72	5.42	11.4
GDP	19	27.42	0.35	26.87	27.98
POP	19	.96	0.13	.81	1.26

Internet adoption exhibits pronounced cross-national variation, with Maldives demonstrating the highest mean penetration (49.06%), followed by Bhutan (38.94%) and Sri Lanka (23.10%). Conversely, Pakistan reports the region's lowest internet usage (12.56%), while India (15.98%) and Bangladesh (16.31%) occupy intermediate positions. Economic indicators highlight India's regional dominance in economic scale (LNGDP = 29.7), while Bhutan (22.82) and Maldives (22.74) represent smaller economies. Inflationary pressures prove most severe in Pakistan (INF = 10.73%) and Sri Lanka (10.24%), contrasting with Maldives' stability (3.71%).

Table 3 presents the correlation matrix of all the variables to test the multicollinearity problem. The reported values for independent variables are truly less than the standard (+ or – 0.70) developed by [67] beyond which the multicollinearity problem occurs.

**Table 3:** Panel correlation matrix

Variables	(1)	(2)	(3)	(4)	(5)	(6)	(7)	(8)	(9)	(10)	(11)	(12)	(13)	(14)	(15)	(16)	(17)	(18)	(19)
HCE	1.00																		
HDI	0.529*	1.00																	
HDIM	0.495*	0.990*	1.00																
HDIF	0.539*	0.984*	0.955*	1.00															
ICT	0.721*	0.620*	0.612*	0.611*	1.00														
IUI	0.604*	0.585*	0.565*	0.602*	0.677*	1.00													
MPS	0.669*	0.681*	0.688*	0.693*	0.733*	0.744*	1.00												
BBS	0.721*	0.618*	0.608*	0.611*	1.000*	0.677*	0.733*	1.00											
FTS	0.207*	0.618*	0.619*	0.602*	0.10	−0.03	0.218*	0.10	1.00										
MM	−0.48*	−0.86*	−0.86*	−0.83*	−0.49*	−0.58*	−0.67*	−0.49*	−0.55*	1.00									
OPHE	−0.53*	−0.49*	−0.46*	−0.60*	−0.41*	−0.57*	−0.37*	−0.41*	−0.21*	0.58*	1.00								
ICTFP	0.669*	0.643*	0.639*	0.631*	0.991*	0.669*	0.759*	0.991*	0.13	−0.52*	−0.38*	1.00							
ICTMP	0.753*	0.594*	0.583*	0.589*	0.995*	0.675*	0.705*	0.995*	0.08	−0.46*	−0.42*	0.973*	1.00						
ICTUP	0.666*	0.659*	0.647*	0.655*	0.988*	0.684*	0.759*	0.988*	0.13	−0.52*	−0.41*	0.996*	0.971*	1.00					
ICTRP	0.658*	0.661*	0.650*	0.655*	0.987*	0.685*	0.763*	0.987*	0.14	−0.53*	−0.41*	0.997*	0.968*	1.000*	1.00				
EF	−0.22*	0.274*	0.281*	0.250*	−0.20*	0.10	−0.06	−0.20*	0.321*	−0.43*	−0.24*	−0.17	−0.22*	−0.15	−0.15	1.00			
INF	−0.27*	−0.14	−0.14	−0.15	−0.15	−0.21*	−0.16	−0.15	0.10	0.09	0.182*	−0.12	−0.17	−0.14	−0.13	−0.10	1.00		
GDP	−0.54*	−0.31*	−0.24*	−0.45*	−0.31*	−0.40*	−0.32*	−0.31*	−0.25*	0.317*	0.784*	−0.291*	−0.32*	−0.33*	−0.33*	−0.03	0.196*	1.00	
POP	0.653*	−0.06	−0.06	−0.08	0.188*	0.10	0.252*	0.188*	−0.04	−0.10	−0.20*	0.17	0.203*	0.13	0.13	−0.25*	−0.14	−0.25*	1.00

***p < 0.01, ** p < 0.05, * p < *0.1*

### Diagnostics

Prior to estimation, we conducted a series of diagnostic tests to ensure the robustness of our results, addressing potential issues inherent in panel data. We employed the Variance Inflation Factor (VIF) to test for multicollinearity, the modified Wald test for heteroskedasticity, the Wooldridge test for serial correlation, and the Breusch-Pagan LM test for cross-sectional dependence. The results, detailed in Appendix A3 (reported for first six models to conserve space), indicate that all VIF values were below the threshold of 4, effectively ruling out multicollinearity. However, the significant *p*-values from the modified Wald and Wooldridge tests confirmed the presence of both heteroskedasticity and serial correlation in our data. It is pertinent to note that the FMOLS and DOLS estimators are specifically designed to control for these exact issues, providing reliable coefficient estimates. Finally, the insignificant p-value from the Breusch-Pagan LM test suggests that our sample is free from cross-sectional dependence.

To establish the order of integration of the variables, we employed two panel unit root tests: the Im-Pesaran-Shin (IPS) test and a Fisher-type test. The results, presented in the Table 4, are consistent across both testing methods.

**Table 4:** Panel data—unit root analysis

**Notations**	**Im Pesaran-Shin**		**Fisher-Type**	**At first diff**
	**At level**	**At first diff**	**At level**	
HCE_i,t_	0.3147	−4.6376^***^	0.6067	−6.9450^***^
HDI_i,t_	1.4876	−5.0371^***^	−0.3293	−6.0740^***^
HDIM_i,t_	2.0802	−5.5171^***^	2.1708	−9.2896^***^
HDIF_i,t_	1.2885	−5.1517^***^	0.9184	−9.0322^***^
ICT_i,t_	5.5137	−2.8845^***^	4.0527	−3.5541^***^
IUI_i,t_	−0.5123	−1.5718^**^	1.2173	−2.7897^**^
MPS_i,t_	1.7810	−3.8076^**^	1.9443	−5.4824^**^
BBS_i,t_	3.6135	−2.9802^***^	3.7589	−3.6961^***^
FTS_i,t_	−1.1291	−4.7372^**^	1.2915	−7.2314^**^
MM_i,t_	−1.2368	−5.7954^***^	−0.8777	−9.6480^***^
OPHE_i,t_	1.6205	−4.4987^***^	1.1167	−7.1000^***^
ICT*MP_i,t_	5.8967	−2.7577^**^	4.2775	−3.3498^**^
ICT*FP_i,t_	5.1613	−3.0133^***^	3.9879	−3.7579^***^
ICT*UP_i,t_	5.5149	−2.7928^***^	4.2123	−3.4268^***^
ICT * RP_i,t_	5.4125	−2.8125^**^	4.1329	−3.4622^**^
EF * RP_i,t_	−1.0407	−5.7441^**^	−1.0382	−10.250^**^
INF_i,t_	−2.3487	−6.0199^***^	−1.0720	−9.4148^***^
GDP_i,t_	1.9143	−5.1718^**^	0.7587	−8.3076^**^
POP_i,t_	−0.5249	−5.1068^**^	1.2076	−6.2029^**^

For all variables, the null hypothesis of a unit root (non-stationarity) cannot be rejected at their levels, as evidenced by the statistically insignificant test statistics. However, the null hypothesis is strongly rejected at the first difference for every variable, as indicated by the highly significant negative statistics (p < 0.01). This confirms that all variables in our study are integrated of order one, I[1]. This property is a essential prerequisite for proceeding with cointegration analysis.

### Cointegration

To examine long-run equilibrium relationships, we employ the panel cointegration approach developed by [62]. Table 5 presents the Kao (Augmented Dicky-fuller test) and Pedroni (Modified Phillips–Perron test) cointegration test results for our models. Cointegration results establishes distinct long-term relationships between ICT and human development indicators across South Asia. For Model 1 (ICT‒HDI) and Model 2 (ICT‒HDIM), results confirm statistically significant cointegration (*p* < 0.05), indicating that digital expansion reinforces both composite human development and male-specific development over extended periods. However, Model 3 (ICT‒HDIF) reveals insignificant long-run relationship, demonstrating that current technological diffusion systematically bypasses female populations. This gender-asymmetric outcome directly contravenes the equity imperative central to Sustainable Development Goal 3, which mandates universal access to health-enabling resources. The absence of cointegrated female advancement signals that passive ICT proliferation cannot overcome structural barriers to women’s digital inclusion. Consequently, South Asian governments must fundamentally redesign ICT policy frameworks. Without such intentional recalibration, technological advancement will remain misaligned with SDG 3’s foundational principle of leaving no one behind.

**Table 5:** Panel data—Kao residual and Pedroni cointegration analyses

Test	Kao Residual		Pedroni	
**Model Specification**	**Test**	**Prob**	**Test**	**Prob**
ICT-HDI	ADF	0.0504	MPP	0.0733
ICT-HDIM	ADF	0.0393	MPP	0.0834
ICTHDIF	ADF	0.1566	MPP	0.1713
ICT-HCE	ADF	0.0430	MPP	0.0216
Channel analysis				
ICT-MM	ADF	0.4804	MPP	0.2424
MM-HDI	ADF	0.0730	MPP	0.0369
ICT-OPHE	ADF	0.0001	MPP	0.0033
OPHE-HDI	ADF	0.0000	MPP	0.0134
Heterogeneity analysis				
ICT*FP-HDI	ADF	0.0009	MPP	0.0137
ICT*MP-HDI	ADF	0.0000	MPP	0.0138
ICT*FP-HCE	ADF	0.0004	MPP	0.0179
ICT*MP-HCE	ADF	0.0000	MPP	0.0164
ICT*RP-HDI	ADF	0.0000	MPP	0.0138
ICT*UP-HDI	ADF	0.0000	MPP	0.0137
ICT*RP-HCE	ADF	0.0000	MPP	0.0177
ICT*UP-HCE	ADF	0.0494	MPP	0.0177

^***^p < 0.01, **p < 0.05,*p < 0.1

Model 4 (ICT-HCE relationships) demonstrate statistically significant cointegration at (p < 0.05), confirming the existence of a long-run association between ICT development and HCE in South Asian economies.

Results from the channel analysis (Models 5–8) confirm that only out-of-pocket health expenditures (OPHE) function as significant mediating pathways between ICT infrastructure and human development outcomes in South Asia. While Model 5 establishes statistically insignificant long-run equilibria between ICT and MM. Furthermore, evidence from Models 6 and 8 reveals robust cointegration between MM and HDI, and OPHE and HDI, respectively. This mediation elucidates the precise mechanisms (OPHE) through which ICT progression enhances human development.

Moderation analysis (Models 9–16) reveals that ICT's developmental contributions are robustly amplified through population-specific interactions. Models 9–12 demonstrate statistically significant (*p* < 0.01) long-term relationships when ICT is moderated by female population (ICTFP) and male population (ICTMP), with both interactions exhibiting cointegration with human development (HDI) and health expenditure (HCE). Similarly, Models 13–16 confirm significant cointegration for ICT moderated by urban (ICTUP) and rural populations (ICTRP) against HDI (*p* < 0.01) and HCE (*p* < 0.05). These uniformly significant results establish two critical insights: First, ICT's developmental impact operates without exception across gender and geographic divides when population-specific adoption patterns are accounted for. Second, the consistent long-term equilibria verify that digital infrastructure functions as a foundational enabler of socioeconomic advancement (captured by HDI) and equitable health financing (reflected in HCE)—core determinants of Quality of Life across South Asia.

### Regression results

Once co-integration in models is confirmed, we proceed to estimate long-run coefficients using the Fully Modified Ordinary Least Square (FMOLS). Table 6 presents the FMOLS regression results elucidating the long-term determinants of human development and health expenditure across South Asia. For Column 1 (HDI as dependent variable), ICT infrastructure demonstrates a statistically significant positive coefficient of 0.0118 (t = 4.71, *p* < 0.01), indicating that a unit increase in digital advancement corresponds to a 0.0118-unit rise in composite human development. Similar findings have been reported by [63, 68]. Economic freedom (EF) also exhibits a positive association (β = 0.00175, t = 8.37), while inflation exerts a detrimental effect (β = −0.00111, t = −5.62). Larger economies (LNGDP) significantly enhance HDI (β = 0.112, t = 14.15), though population growth presents a paradoxical positive relationship (β = 0.0135).

**Table 6:** Baseline Regression Estimation

Column	(1)	(2)	(3)
**Dep. Var**	**HDI**	**HDIM**	**HCE**
ICT	0.0118^***^	0.0119^***^	6.008^***^
	(4.71)	(3.79)	(9.37)
EF	0.00175^***^	0.00188^***^	−0.330^***^
	(8.37)	(7.17)	(−5.79)
INF	−0.00111^***^	−0.00125^***^	0.453^***^
	(−5.62)	(−5.06)	(11.14)
LNGDP	0.112^***^	0.0940^***^	41.12^***^
	(14.15)	(9.45)	(20.66)
POPG	0.0135^***^	0.0219^***^	−5.667^***^
	(3.03)	(3.92)	(−3.22)
Constant	−2.565^***^	−2.051^***^	−1075.0^***^
	(−12.38)	(−7.89)	(−21.06)
*R*^2^	0.991	0.982	0.993
adj. *R*^2^	0.987	0.974	0.989

^***^p < 0.01, **p < 0.05,*p < 0.1 (t-statistics are reported in parenthesis)

Column 2 (HDIM) reveals analogous directional patterns, with ICT again emerging significant (*p* < 0.01), revealing strong impact of ICT on gender-specific HDI growth. In Column 3 ICT has a positive and significant effect on HCE (6.008, t = 9.37). This positives association signifies that digital infrastructure expansion induces substantial long-term investments in healthcare systems across South Asia [63, 69]. This relationship likely operates through multiple channels: enhanced telemedicine adoption, digital health record integration, and technology-driven diagnostic capabilities which collectively increase healthcare service utilization and necessitate higher systemic funding.

In Summary, ICT’s positive effects on HDI and HCE reveal a dual pathway through which digital advancement elevates Quality of Life (QoL). The HDI linkage demonstrates ICT’s role in amplifying socioeconomic capabilities—education, income, and longevity—while the HCE relationship confirms its function in strengthening the health financing foundations underpinning population wellbeing in developing South Asian nations.

#### Heterogeneity analysis

Table 7 presents the gender-disaggregated moderation analysis. The empirical outcome reveals nuanced differentials in how ICT diffusion benefits male and female populations, with implications for equitable development. Both female-oriented (ICTFP) and male-oriented (ICTMP) ICT expansion demonstrate statistically significant positive effects on human development (HDI) and health expenditure (HCE) at the 1% significance level (*p* < 0.01). Although, in the health financing domain, both gender pathways exhibit identical coefficients for HCE (β = 0.120), indicating gender-neutral monetary investment returns per unit of ICT advancement. However, for HDI, ICTMP (β = 0.000246) exerts a 7.5% stronger influence than ICTFP (β = 0.000227), suggesting that digital infrastructure currently delivers marginally greater socioeconomic benefits to male populations through enhanced access to education, economic opportunities, and skill development [53]. This divergence paradoxically coexists with our earlier finding of no long-term cointegration between ICT and HDIF (Table 5), revealing that while ICT improves women’s access to aggregate development outcomes in South Asian countries, it fails to dismantle structural constraints to gender equity in health and education—a critical deficit against Sustainable Development Goal 3’s equity mandate.

**Table 7:** Gender-disaggregated heterogeneity analysis

Column	(1)	(2)	(3)	(4)
**Dep. Var**	**HDI**	**HDI**	**HCE**	**HCE**
ICTFP	0.000227^***^		0.120^***^	
	(5.65)		(24.47)	
ICTMP		0.000246^***^		0.120^***^
		(5.94)		(20.62)
EF	0.00174^***^	0.00175^***^	−0.317^***^	−0.343^***^
	(10.18)	(10.40)	(−14.15)	(−13.58)
INF	−0.00111^***^	−0.00110^***^	0.450^***^	0.455^***^
	(−6.94)	(−6.88)	(28.59)	(24.95)
GDP	0.113^***^	0.111^***^	40.83^***^	41.41^***^
	(17.62)	(17.23)	(52.75)	(46.48)
POPG	0.0133^***^	0.0136^***^	−5.387^***^	−5.954^***^
	(3.68)	(3.75)	(−7.85)	(−7.59)
Constant	−2.593^***^	−2.539^***^	−1068.3^***^	−1082.0^***^
	(−15.45)	(−15.04)	(−53.86)	(−47.32)
*R*^2^	0.991	0.991	0.993	0.993
adj. *R*^2^	0.987	0.987	0.989	0.989

*t* statistics in parentheses, ^*^p < 0.1, ^**^p < 0.05, ^***^p < 0.01

Table 8 reveals geographic disparities on how ICT-driven development manifests across South Asia. When examining urban populations (ICTUP), results demonstrate a statistically significant positive influence on both human development (HDI: β = 0.000856, *p* < 0.10) and healthcare expenditure (HCE: β = 0.355, *p* < 0.01). This urban advantage likely stems from concentrated digital infrastructure, specialized workforce availability, and integrated service ecosystems that amplify technology's developmental returns. Furthermore, ICT moderated through rural populations (ICTRP) also exhibits significant effects on HDI (β = 0.000776, *p* < 0.10) and HCE (β = 0.323, *p* < 0.01), though with marginally diminished magnitude. The 7.3% smaller coefficient for rural ICT influence on HDI and 9.0% reduction for HCE reflect persistent challenges, such as limited last-mile connectivity, weaker technology absorption capacity [53], and fragmented healthcare access in remote regions. Moreover, digital divide significantly widens the gap between urban and rural areas, intensifying inequities in access to technology, information, and digital skills. Urban regions typically enjoy advanced digital infrastructure and high-speed internet, whereas rural populations frequently face obstacles in utilizing online education, e-commerce, and other digital resources [70]. This gradient effect risks deepening spatial health inequities, a fundamental challenge to SDG 3’s principle of leaving no one behind.

**Table 8:** Geographic-disaggregated heterogeneity analysis

Column	(1)	(2)	(3)	(4)
**Dep. Var**	**HDI**	**HDI**	**HCE**	**HCE**
ICTUP	0.000856^*^		0.355^***^	
	(1.81)		(10.64)	
ICTRP		0.000776^*^		0.323^***^
		(1.62)		(9.80)
EF	0.00207^***^	0.00203^***^	−0.297^***^	−0.333^***^
	(2.92)	(2.77)	(−5.54)	(−6.15)
INF	−0.00116^*^	−0.00113	0.447^***^	0.453^***^
	(−1.75)	(−1.63)	(11.89)	(11.73)
LNGDP	0.0974^***^	0.0995^***^	40.02^***^	41.24^***^
	(3.64)	(3.57)	(21.53)	(21.81)
POPG	0.0190	0.0170	−5.025^***^	−5.732^***^
	(1.27)	(1.08)	(−3.07)	(−3.43)
Constant	−2.185^***^	−2.238^***^	−1047.1^***^	−1078.1^***^
	(−3.12)	(−3.07)	(−21.99)	(−22.23)
*R*^2^	0.993	0.994	0.993	0.993
adj. *R*^2^	0.990	0.991	0.989	0.989

*t* statistics in parentheses, ^*^p < 0.1, ^**^p < 0.05, ^***^p < 0.01

For policymakers, bridging this urban–rural efficacy gap remains imperative for achieving universal health coverage targets across South Asia’s heterogeneous landscape.

**Fig. 6:**
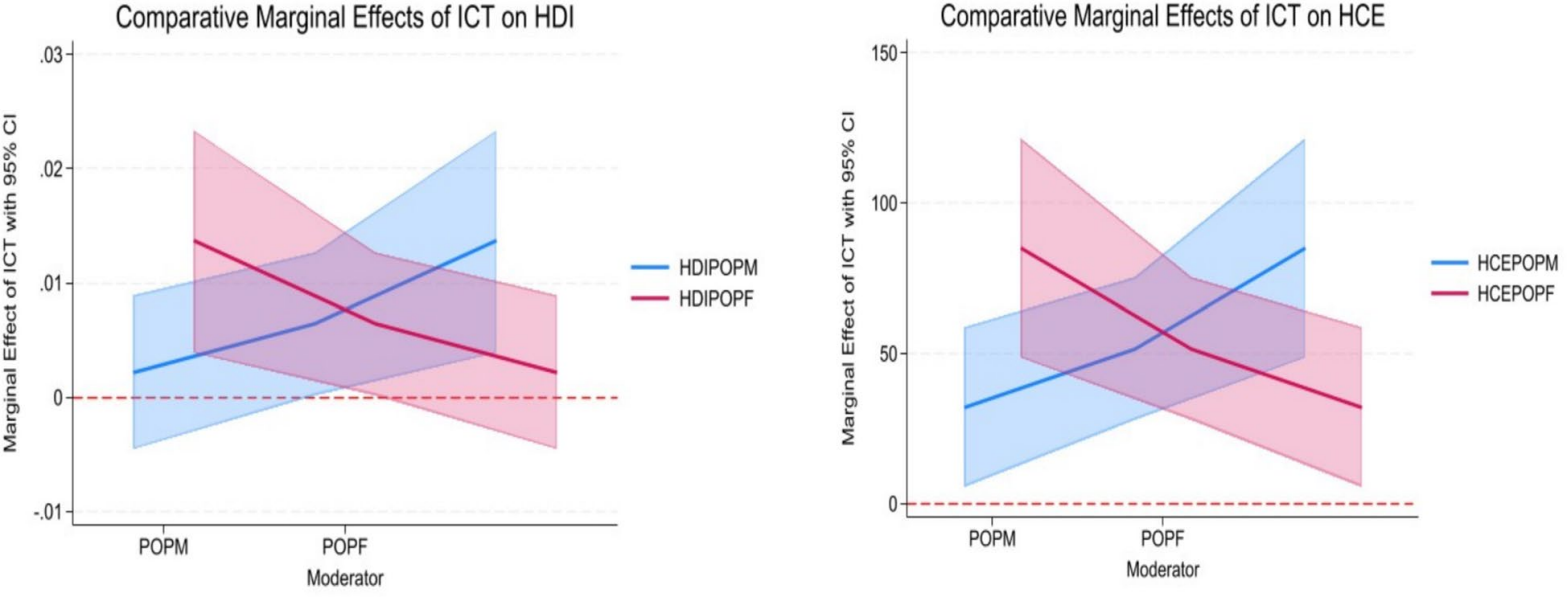
comparative marginal effects of ICT on HDI and HCE for male and female population

**Fig. 7:**
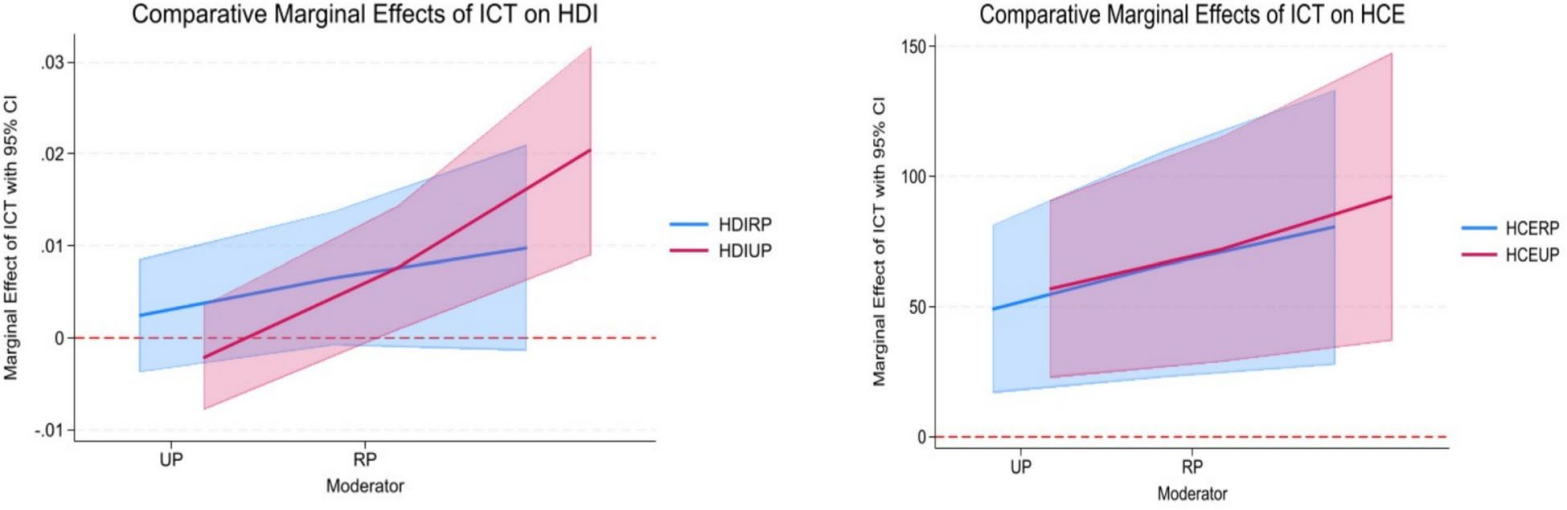
comparative marginal effects of ICT on HDI and HCE for urban and rural population

The comparative marginal effects plots (Fig. 6 & 7) provide visual confirmation of the core findings from our regression analyses in Tables 7 and 8, illustrating the asymmetric impact of ICT across gender and geography. Figure 6 demonstrate a consistent and pronounced gender gap. The marginal effect of ICT on both HDI and HCE is significantly larger when moderated through the male population (POPM) compared to the female population (POPF). The line for HDIPOPM/HCEPOPM is notably higher than for HDIPOPF/HCEPOPF, with their confidence intervals likely showing minimal overlap. This visual asymmetry directly aligns with the higher coefficients for ICTMP reported in Table 7, confirming that male populations are significantly more effective at converting ICT infrastructure into tangible developmental and health investment gains.

Similarly, Fig. 7 offer clear visual evidence of the urban–rural digital divide. The marginal effect of ICT is stronger for urban population (UP) versus a rural one (RP). More precisely, the line for HCEUP is higher than for HCERP, a pattern that is mirrored in the HDI graph. This provides graphical support for the regression results in Table 8, where the coefficient for ICTUP was larger than for ICTRP, underscoring that urban areas are more advantaged in leveraging ICT for improving development outcomes and health financing.

#### Mechanistic analysis

Table 9 present the results of our mediation analysis, based on cointegration results we have not included ICT-MM for our regression analysis. Wile, FMOLS results establish two distinct pathways through which healthcare system dynamics influence human development. First, reductions in maternal mortality (MM) exhibit a statistically significant positive effect on human development (β = −0.000044, *p* < 0.01), confirming that lower maternal deaths contribute robustly to societal wellbeing. This relationship underscores maternal survival as a fundamental indicator of well-being [71].

**Table 9:** Mechanism analysis

Column	(1)	(2)	(3)
**Dep. Var**	**HDI**	**OPHE**	**HDI**
ICT	0.0140^***^	3.622^***^	0.0210^***^
	(10.10)	(6.90)	(9.99)
MM	−0.000044^***^		
	(−3.61)		
OPHE			0.00119^***^
			(6.21)
EF	0.00149^***^	0.290^***^	0.00274^***^
	(11.82)	(6.21)	(14.78)
INF	−0.000834^***^	−0.115^***^	−0.000925^***^
	(−6.71)	(−3.45)	(−7.20)
LNGDP	0.0974^***^	−5.056^***^	0.0773^***^
	(17.22)	(−3.10)	(12.34)
POPG	0.0110^***^	6.160^***^	0.0597^***^
	(4.75)	(4.27)	(10.67)
Constant	−2.139^***^	189.1^***^	−1.790^***^
	(−13.64)	(4.52)	(−11.00)
*R*^2^	0.992	0.837	0.993
adj. *R*^2^	0.987	0.746	0.987

*t* statistics in parentheses, ^*^p < 0.1, ^**^p < 0.05, ^***^p < 0.01

Results of column 2 and 3 indicates that ICT expansion significantly increases out-of-pocket health expenditures (OPHE) (β = 3.622, *p* < 0.01), that subsequently improves the HDI (β = 0.00119, *p* < 0.01). This suggests households may accept immediate financial burdens to access previously unavailable digital health services, translating into long-term human capital gains. Ngepah and Ndzignat Mouteyica [59] also reported positive influence of ICT on out-of-pocket health expenditures in 40 African countries. However, this pathway raises concerns about financial sustainability, as heightened OPHE contradicts Sustainable Development Goal 3.8’s objective of reducing catastrophic health spending.

### Robustness test

Table 10 presents the results of the Dynamic Ordinary Least Squares (DOLS) estimation, reported for core variables to conserve space. The findings robustly confirm the baseline FMOLS results: ICT exhibits a statistically significant and positive relationship with the Human Development Index (HDI), male HDI (HDIM), and healthcare expenditure (HCE) in South Asia.

**Table 10:** Panel dynamic ordinary least square analyses results

**Baseline regression analyses**				
Model	(1)	(2)	(3)	(4)
	HDI	HDIM	HDIF	HCE
ICT	0.071^***^	0.063^***^	––-	52.2^***^
	(25.88)	(15.88)	––-	(11.86)
Gender dis-aggregated moderation analyses				
Model	(5)	(6)	(7)	(8)
	HDI	HDI	HCE	HCE
ICTMP	0.00115^***^		0.790^***^	
	(30.70)		(10.45)	
ICTFP		0.00098^***^		0.711^***^
		(28.13)		(10.11)
Geographically dis-aggregated moderation analyses				
Model	(9)	(10)	(11)	(12)
	HDI	HDI	HCE	HCE
ICTUP	0.00383^***^		2.560^***^	
	(32.27)		(10.55)	
ICTRP		0.00339^***^		2.306^***^
		(31.31)		(10.47)
Mechanism analyses				
Model	(13)	(14)	(15)	(16)
	MM	HDI	OPHE	HDI
MM	––-	−0.00796^***^		
	––-	(−23.32)		
ICT			−7.817^***^	
			(−3.28)	
OPHE				−0.00406^***^
				(−28.39)

*t* statistics in parentheses, ^*^p < 0.1, ^**^p < 0.05, ^***^p < 0.01

Further reinforcing the primary analysis, the disaggregated results show that the benefits of ICT are not uniformly distributed. The coefficients for ICT interaction with male (ICTMP) and urban (ICTUP) populations are larger than those for female (ICTFP) and rural (ICTRP) populations, respectively. This confirms that males and urban residents derive greater advantages from ICT diffusion.

The analysis also reaffirms that reducing maternal mortality is a positive and significant contributor to HDI. However, a notable divergence from the FMOLS results emerged: out-of-pocket health expenditures (OPHE) show a significant negative association with HDI under the DOLS specification. This suggests that higher OPHE may impede human development progress in the region, a finding that warrants further investigation.

Finally, to ensure our results are not driven by outliers, we re-estimated the models excluding Maldives, a country with exceptionally high per-capita health spending. The results (available upon request) remain consistent, underscoring the robustness of our core findings.

## Conclusion and implications

The pursuit of Sustainable Development Goals (SDGs) in developing nations hinges critically on improving healthcare access, advancing human development, and reducing maternal mortality. These interconnected priorities lie at the heart of inclusive progress—where better health systems strengthen economic productivity, education, and equity, while higher HDI scores reflect broader societal wellbeing. Maternal mortality remains a stark indicator of healthcare inequality, with high rates signaling systemic failures in women’s access to medical services. In South Asia, where socioeconomic disparities persist, accelerating progress toward SDG 3 (good health and wellbeing) and SDG 10 (reduced inequality) demands targeted policies that leverage technology while addressing structural inequities.

This study examined the impact of information and communication technology (ICT) on human development index and healthcare expenditures across seven South Asian economies from 2005 to 2023. Applying FMOLS and DOLS to address non-stationarity, we establish that ICT significantly drives aggregate improvements in quality of life (proxied by HDI and HCE). However, heterogeneity analyses reveal three significant disparities influenced by factors such as gender and geography.

First, ICT’s positive influence on overall development is unequivocal, with significant long-term effects on both HDI and health investment. However, this progress masks persistent gender inequities: while ICT influences male versus female human development (HDIM and HDIF), with notably weaker (insignificant) benefits for women highlights systemic exclusion. The persistence of this disparity in South Asia is further strengthened when our mediation analysis reveals insignificant long-run association between ICT developments and maternal mortality. Second, ICT enhances male development (ICTMP → HDI: β = 0.000246) 7.5% more strongly than female development (ICTFP → HDI: β = 0.000227). These disparities highlight that ICT developments impede SDG 10 (reduced inequality) in South Asia, indicating that digital growth alone cannot dismantle structural barriers to women’s advancement.

Third, our analysis further confirms pronounced geographic inequities in ICT's developmental returns. Empirical evidence reveals that urban-contextualized ICT diffusion (ICTUP) generates 7.3% stronger improvements in human development (HDI) and 9.0% greater increases in health expenditures (HCE) compared to rural-oriented diffusion (ICTRP). This quantitatively substantiated urban bias reflects that digital divide significantly widens the gap between urban and rural areas, intensifying inequities in access to technology, information, and digital skills. Urban regions typically enjoy advanced digital infrastructure and high-speed internet, whereas rural populations frequently face obstacles in utilizing digital resources [70]. Such spatial imbalances directly undermine the equitable distribution of quality-of-life advancements across South Asia's development landscape.

Finally, our mediation path analysis reveals important trade-offs. Although, ICT does not contribute to maternal mortality in South Asia. However, reducing MM directly improves human development (β = −0.000044, *p* < 0.01), confirming better maternal health strengthens societal wellbeing. Conversely, ICT expansion exerts a strong positive influence on out-of-pocket health expenditures (β = 3.622, *p* < 0.01), which paradoxically associates with HDI gains (β = 0.00119, *p* < 0.01). This path analysis exerts that investments in digital health technologies initially increase household medical spending probably through new costly ICT enabled services like telemedicine and health applications. While this creates temporary financial pressure, such spending ultimately supports better health access and outcomes, contributing to long-term quality of life improvements. This tension between short term costs and long-term gains presents challenges for SDG 3.8, which aims to protect families from medical financial hardship while ensuring universal health coverage.

In summary, the relationship between ICT and well-being is complex and non-linear for South Asian countries. With studies reporting that nearly 70% of ICT projects fail [72], poorly designed initiatives, particularly those lacking inclusivity, technical training, or adaptability, risk counterproductive outcomes. To mitigate these risks and maximize socioeconomic benefits, regionally tailored policies are essential. South Asia continues to face significant challenges in achieving the SDGs. This is evidenced by the region's overall poor performance in global rankings, with countries clustered in the bottom half: India (99th), Pakistan (140th), Bangladesh (114th), Sri Lanka (93rd), Bhutan (74th), Nepal (85th), and the Maldives (53rd). This trend of underperformance is particularly acute for SDG 3 (Good Health and Well-being), where India, Pakistan, Bangladesh, and Nepal face "major challenges," while "significant challenges remain" for Sri Lanka, Bhutan, and the Maldives [73]. Therefore, drawing on the empirical findings, policy must be precisely targeted to address the specific asymmetries revealed. First, the 7.5% efficacy gap in how ICT benefits male versus female human development mandates a shift from generic digital access to gender-responsive ICT initiatives, such as subsidized devices and digital literacy programs specifically for women, to dismantle the structural barriers that prevent HDIF cointegration (advancing SDG 5). Second, to bridge the 7.3% and 9.0% urban–rural gaps in HDI and health expenditure impact, policy must prioritize rural digital infrastructure with a health mandate, investing in last-mile broadband and mobile health clinics to contextualize ICT for underserved areas. Third, since ICT increases out-of-pocket health spending which then mediates HDI gains, ICT-linked health financing safeguards like telemedicine-covered insurance are essential to mitigate financial risk while preserving access (supporting SDG 3). Finally, the insignificant direct link between ICT and maternal mortality necessitates a dual-pronged strategy that couples digital tools like diagnostic AI with direct investments in physical maternal care upgrades to leverage the indirect HDI benefits from reduced mortality. Collectively, these actions address the quantified disparities to ensure ICT functions as an engine of inclusive sustainable development in South Asia.

This research also highlights several limitations that can serve as potential opportunities for researchers to determine their future research directions. First, we have analysed the role of ICT on the quality of life in South Asian countries with several mediation and moderating roles. However, these countries possess diverse cultural and religious values and beliefs that could influence this association. Country-specific analyses could yield valuable insights. Second, a pre- and post-COVID-19 analyses could clarify whether the pandemic amplified or diminished ICT’s role in well-being. Finally, future studies could also expand beyond traditional ICT measures to examine advanced digital technologies like AI adoption, blockchain integration, and IoT penetration, which may reveal new pathways for ICT-driven development in emerging economies.

## Data Availability

The datasets used and/or analysed during the current study are available from the corresponding author on reasonable request.

## Appendix 1

**Table 11:** PCA eigenvalues

**Component**	**Eigenvalue **	**Difference**	**Proportion**	**Cumulative**
Comp1	2.427	2.104	0.809	0.809
Comp2	0.323	0.074	0.107	0.917
Comp3	0.248	.	0.082	1.000

## Appendix 2

**Table 12:** PCA factor loadings

Variable	Comp1	Comp2	Comp3	Unexplained
IUI	0.572	0.711	0.408	0
BBS	0.572	-0.702	0.422	0
MPS2	0.587	-0.007	-0.809	0

## Appendix 3

**Table 13:** Diagnostic tests

VIF analysis		Modified Wald test		Wooldridge test		Breusch-Pagan LM test	
Variable	VIF	Model 1	P= 0.00	Model 1	P= 0.00	Model 1	Pr = 0.77
ICT	1.37	Model 2	P= 0.00	Model 2	P= 0.01	Model 2	Pr = 0.87
LNGDP	1.35	Model 4	P= 0.00	Model 4	P= 0.00	Model 4	Pr = 0.19
POPG	1.18	Model 5	P= 0.00	Model 5	P= 0.00	Model 5	Pr = 0.60
EF	1.13	Model 6	P= 0.00	Model 6	P= 0.00	Model 6	Pr = 0.61
INF	1.11	Model 7	P= 0.00	Model 7	P= 0.00	Model 7	Pr = 0.19
